# 3D-printed oral stents improve setup stability, geometric morphology, and dosimetric outcomes while reducing functional impairment in radiotherapy for nasopharyngeal carcinoma

**DOI:** 10.3389/fonc.2026.1881998

**Published:** 2026-08-14

**Authors:** Xiaozhou Zeng, Xuhui Zhang, Zhongfan Liao, Jirui Wang

**Affiliations:** Department of Tumor Radiotherapy, Sichuan Provincial People’s Hospital, School of Medicine, University of Electronic Science and Technology of China, Chengdu, China

**Keywords:** cone-beam CT, geometric reproducibility, intraoral stent, nasopharyngeal carcinoma, radiotherapy, setup error, toxicity

## Abstract

**Objective:**

To evaluate whether personalized three-dimensional (3D)-printed intraoral stents improve functional outcomes and setup reproducibility in nasopharyngeal carcinoma (NPC) radiotherapy without compromising dosimetric fidelity.

**Methods:**

This retrospective study included 99 NPC patients treated with definitive VMAT between January 2023 and December 2024. Patients were divided into stent group (n = 48) who wore 3D-printed intraoral stents during radiotherapy, and control group (n = 51) who received no intraoral device. Radiation-induced toxicities (xerostomia, dysgeusia, dysphagia, hearing loss) were assessed via telephone questionnaire. Interfractional setup reproducibility of organs at risk (OARs) was evaluated using daily cone-beam CT, and dosimetric parameters (D_2_, D_98_, Dmean) were compared between groups.

**Results:**

Stent use was significantly associated with reduced incidence and severity of hearing loss, dysgeusia, xerostomia, and dysphagia (all P < 0.05). Interfractional geometric variability of parotids and tongue was significantly reduced in the stent group (P < 0.05), indicating improved geometric stability. *Post-hoc* dosimetric analysis revealed significantly lower inner ear doses (Dmean/Dmax) in the stent group (P < 0.05), providing a dosimetric basis for hearing preservation.

**Conclusion:**

Patient-specific 3D-printed intraoral stents confer clinically meaningful benefits in NPC radiotherapy by improving functional preservation and interfractional setup reproducibility. These benefits appear driven by two complementary mechanisms: direct dose sparing of fixed OARs (inner ear) and enhanced geometric stabilization of mobile OARs (tongue, parotids). These findings support integrating such stents into routine practice to reduce toxicity without compromising planned dose delivery.

## 1 Introduction

Nasopharyngeal carcinoma (NPC), which is highly prevalent in southern China and Southeast Asia, remains a unique head and neck malignancy characterized by its anatomy (1). Radiotherapy remains the primary treatment modality in NPC, with wide adoption of intensity-modulated radiotherapy (VMAT) significantly improving locoregional control rates as well as overall patients’ survival (2, 3); however, radiation-induced toxicities (including xerostomia, dysphagia, taste impairment, hearing loss and temporomandibular dysfunction) still greatly impact the patient’s quality of life (4).

**Figure 1:**
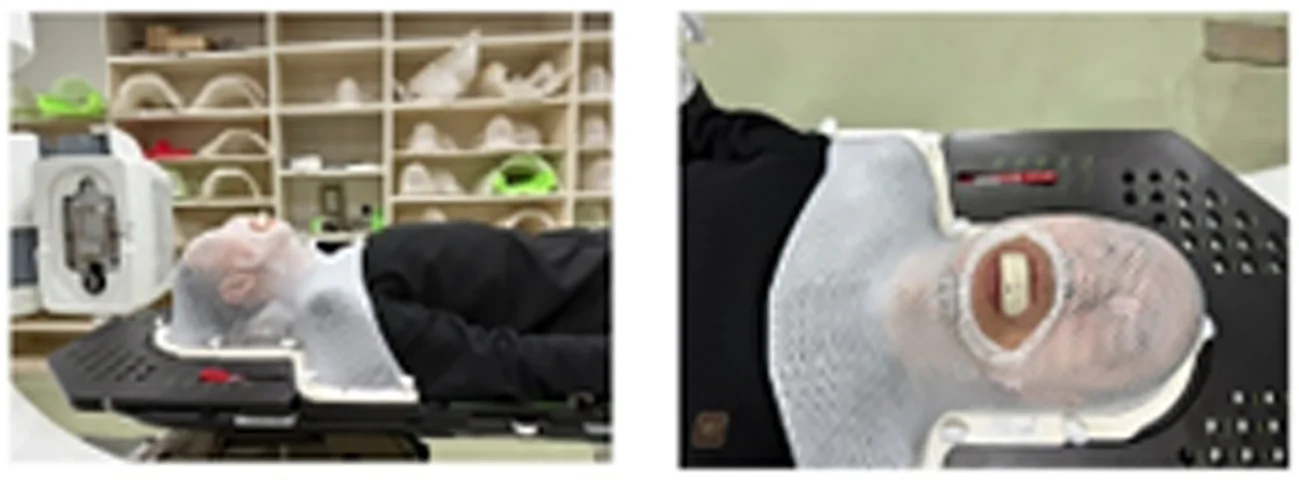
Individualized 3D-printed intraoral stent used during radiotherapy for nasopharyngeal carcinoma.

In recent years, three-dimensional (3D) printing has enabled the fabrication of patient-specific intraoral appliances. Compared with conventional wax- or silicone-based oral stents, patient-specific 3D-printed intraoral stents provide superior customization, reproducibility, and patient comfort. By mechanically stabilizing the tongue and mandibular position, these devices increase the distance between planning target volumes (PTVs) and adjacent organs at risk (OARs), thereby potentially reducing unnecessary radiation exposure to normal tissues (5). Most previous studies have focused on optimizing radiation dose distributions to improve treatment outcomes and reduce late toxicities after NPC radiotherapy, whereas comparatively less attention has been paid to preventing treatment-related toxicities through improved anatomical stabilization during treatment delivery (6).Also, most prior reports were limited to phantom studies, small case series, or mixed tumor sites, with few targeting NPC or assessing long-term functional outcomes (**Supplementary Table 3**). Accordingly, we conducted this comprehensive evaluation in an NPC cohort, emphasizing setup reproducibility, regional tongue motion, and functional toxicity. During each treatment session, patients position the individualized intraoral stent according to dental occlusion and tongue position (7), allowing reproducible anatomical positioning throughout radiotherapy (**Figure 1**).

Previous works demonstrated that intraoral stents could effectively reduce radiation exposure to oral soft tissues during head and neck radiotherapy (8, 9). Recent studies have extended this concept using 3D-printed patient-specific devices, which are generally well tolerated, provide reproducible maxillomandibular positioning, and reduce fabrication time and costs (10, 11). However, the current evidence remains limited in several aspects. First, most published studies have included heterogeneous head and neck cancer populations rather than NPC specifically, and convincing evidence supporting their effectiveness in preventing radiation-induced oral toxicities is still insufficient. Second, these studies have primarily focused on planned dosimetric comparisons, with little attention to interfractional setup reproducibility or systematic evaluation of acute functional toxicities. Third, conventional toxicity grading systems, including the RTOG/EORTC and CTCAE criteria, generally evaluate the tongue as a single homogeneous organ, potentially masking regional dose–response relationships (12, 13) despite the distinct embryological, neural, and functional heterogeneity across tongue subregions (14, 15). Anatomically and functionally, the anterior tongue is primarily responsible for taste and articulation, and is thus closely associated with dysgeusia and speech impairment; the middle tongue plays a critical role in mastication and swallowing; and the posterior tongue, adjacent to the retropharyngeal lymph node target volumes, is at highest risk of incidental radiation exposure and severe late swallowing dysfunction. These regional differences suggest that evaluating the tongue as a single organ may obscure the specific benefits of intraoral stents on individual functional subregions. Therefore, in the present study we performed a subregional analysis of the tongue to better characterize interfractional setup reproducibility and its potential association with functional outcomes.

**Figure 2:**
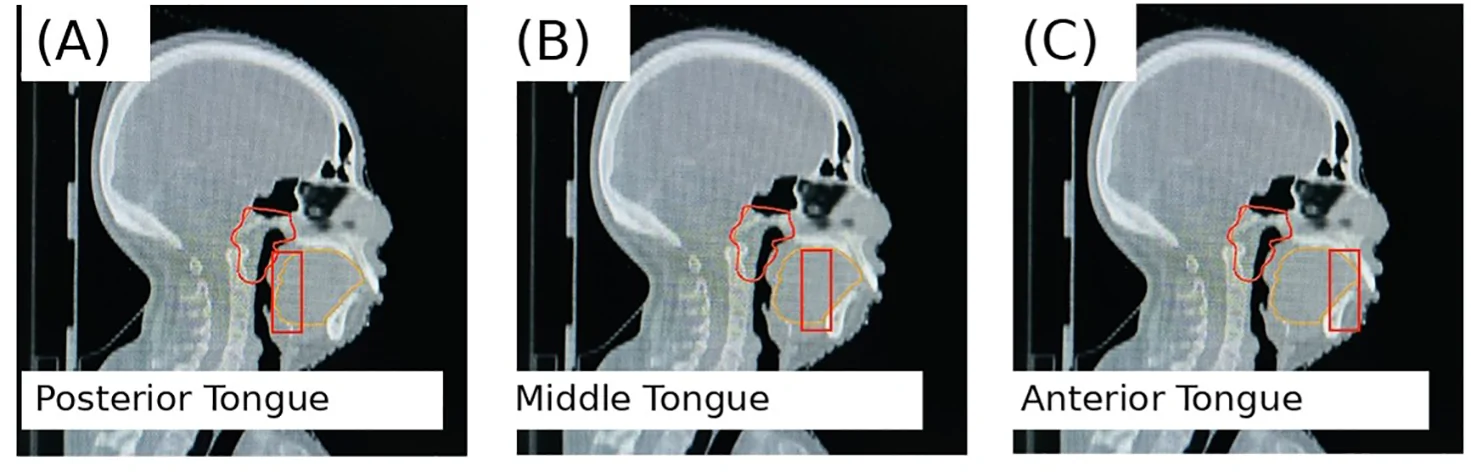
Offline CBCT registration of tongue subregions in the control group during radiotherapy for nasopharyngeal carcinoma **(A)** Posterior tongue (tongue base); **(B)** middle tongue; **(C)** anterior tongue (tongue tip).

**Figure 3:**
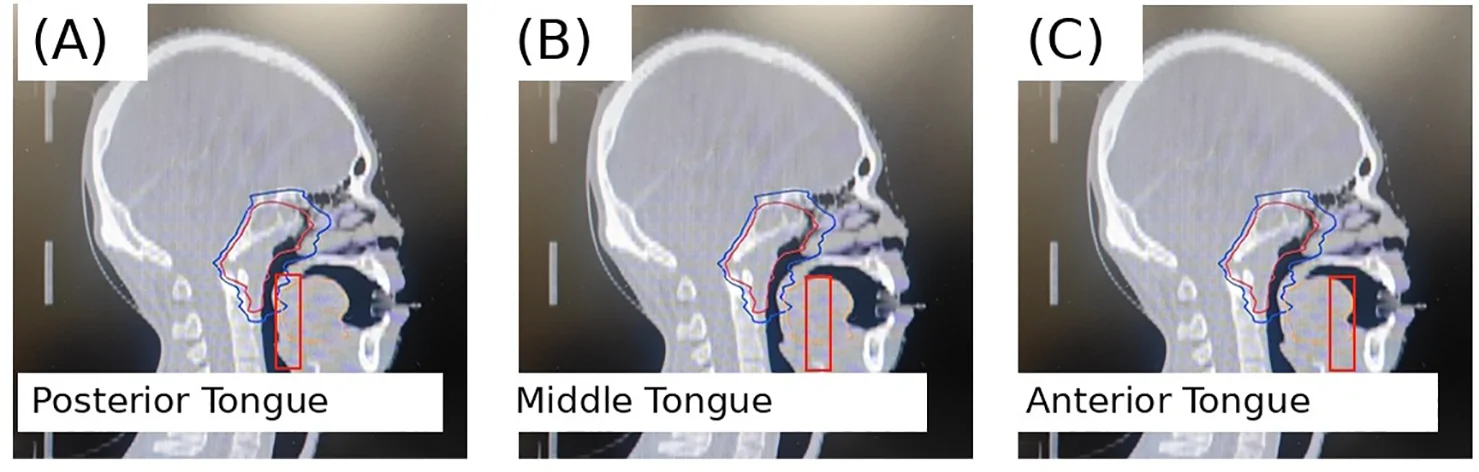
Offline CBCT registration of tongue subregions in the stent group during radiotherapy for nasopharyngeal carcinoma **(A)** Posterior tongue (tongue base); **(B)** middle tongue; **(C)** anterior tongue (tongue tip).

To address this issue, we propose a subregional analysis of the tongue by dividing it into the anterior (tip), middle, and posterior (base) compartments to better characterize interfractional setup reproducibility and its potential association with functional outcomes (16, 17), as illustrated in **Figures 2** and **3**, respectively. Accordingly, we conducted a retrospective cohort study to evaluate the clinical and geometric effects of individualized 3D-printed intraoral stents in patients with NPC undergoing VMAT. Specifically, we compared (1) radiation-induced toxicities, (2) interfractional setup reproducibility of organs at risk, (3) dosimetric parameters, and (4) tongue subregional motion and its potential relationship with functional outcomes. We hypothesized that individualized 3D-printed intraoral stents would improve functional preservation and interfractional setup reproducibility without compromising target dose delivery, thereby providing additional insight into the geometric and functional mechanisms underlying toxicity reduction in NPC radiotherapy.

## 2 Materials and methods

### Study design and patients

This retrospective study was approved by the Institutional Review Board (IRB) of Sichuan Provincial People’s Hospital (Approval No (20264693).). All procedures performed in this study were in accordance with the 1964 Helsinki Declaration and its later amendments. Written informed consent was obtained from all patients prior to study enrollment. This retrospective analysis consecutively enrolled 99 individuals with histologically verified nasopharyngeal carcinoma (NPC) who received definitive radiotherapy using Volumetric Modulated Arc Therapy (VMAT) at the Cancer Center of Sichuan Provincial People’s Hospital from January 2023 to December 2024. All data were derived from routine clinical practice and extracted from the institutional electronic medical record (EMR) system. All patients received two cycles of induction chemotherapy with paclitaxel and cisplatin (TP regimen), followed by definitive radiotherapy. Concurrent cisplatin monotherapy was administered as a radiosensitizer during radiotherapy, with a total of two cycles of concurrent chemotherapy completed.

Participants were categorized into two cohorts according to whether they received personalized 3D-printed intraoral stents during radiotherapy: the stent cohort (n = 48) and the control cohort (n = 51). Patients in the stent group wore the intraoral stent throughout the entire course of radiotherapy, whereas those in the control group did not use any intraoral immobilization or separation device.

Participants were eligible if they: (1) had pathologically diagnosed NPC; (2) were 18–80 years old; (3) had finished the entire radiotherapy regimen; (4) had completed the entire radiotherapy regimen and had at least one post-treatment follow-up record encompassed; and (5) were capable of participating in follow-up via telephone.

Exclusion criteria included: (1) prior head and neck radiotherapy or concurrent head and neck malignancies; (2) severe maxillofacial deformities or prior maxillofacial surgery; (3) neurological or psychiatric disorders affecting the reliability of questionnaire responses; and (4) incomplete follow-up data or withdrawal during follow-up.

The decision to use a personalized 3D-printed intraoral stent was made by the treating physician in consultation with the patient, based on the patient’s oral cavity condition, financial affordability, and personal preference. As this was a retrospective study, patients were not randomly assigned to the stent or control groups. To minimize potential selection bias, multivariable regression analyses were performed to adjust for baseline characteristics. The baseline demographic and clinical characteristics of the study population are summarized in **Table 1**.

**Table 1:** Baseline characteristics of the study population.

Variable	Overall (N=99)	Control group (n=51)	Stent group (n=48)	P value
Gender, n (%)				0.687†
Male	64 (64.6)	31 (60.8)	33 (68.7)	
Female	35 (35.4)	20 (39.2)	15 (31.3)	
Age (years), mean ± SD	55.8 ± 10.7	54.1 ± 11.9	57.7 ± 9.1	0.094†
Chemotherapy, n (%)				—
Yes	0 (0.0)	0 (0.0)	0 (0.0)	
No	99 (100.0)	50 (100.0)	49 (100.0)	
T stage, n (%)				0.105†
T1	0 (0.0)	0 (0.0)	0 (0.0)	
T2	0 (0.0)	0 (0.0)	0 (0.0)	
T3	75 (54.5)	40(84.3)	32(66.7)	
T4	24 (45.5)	11 (15.7)	16 (33.3)	
N stage, n (%)				0.354††
N0	3 (0.0)	1 (1.96)	2 (4.17)	
N1	2 (0.0)	1 (1.96)	1 (2.08)	
N2	90 (0.0)	48 (94.2)	42 (87.5)	
N3	4 (0.0)	1 (1.96)	3 (6.25)	
Radical conventional fractionation (69–73 Gy, 33–35 fx)	99 (100.0)	50 (100.0)	49 (100.0)	0.324 †††
Follow-up time, median (IQR), months	15.5 (12.5–18.5)	15.0 (12.0–18.0)	16.0 (13.0–19.0)	

†P values for categorical variables were calculated using the Chi-square test. b) ††P values for N-stage distribution were calculated using Fisher’s exact test. c) †††P value for Follow-up time was calculated using Mann-Whitney U test

### Fabrication of the 3D-printed oral stent

Patients were placed in the supine position and immobilized on an Orfit all-in-one fixation frame mounted on a Siemens CT simulator (model SOMATOM Definition AS). During positioning, the patient’s midsagittal plane was aligned with the treatment couch midline and the laser-defined midsagittal line, and the acanthiomeatal line was set perpendicular to the couch surface. Both upper limbs were naturally positioned alongside the torso. Before scanning, all metal objects and accessories within the head-and-neck irradiation field were removed, and excessively long hair was trimmed short.

During data acquisition, all patients were required to bite a standardized module with a fixed thickness of 2cm, which maintained a vertical mouth opening of 2cm between the upper and lower dentition. At the same time, the patient was asked to actively press the tongue against the lower alveolar ridge to displace the tongue root posteriorly. CT scanning was then performed using a head-and-neck protocol, with the patient in the supine position and head-first. The scan range extended from 5mm above the vertex to the lower margin of the fifth thoracic vertebra, with a slice thickness of 0.3mm.

The acquired DICOM data were transferred to a 3D-printing facility. The material selection was based on a comprehensive evaluation of the physical, mechanical, and dosimetric properties of polylactic acid (PLA) in comparison with other commercially available materials, as detailed in **Supplementary Table 4**. The stents were fabricated using fused deposition modeling (FDM) technology, with a layer thickness of 0.32mm and the internal filling rate was 12%. The radiation transmittance was 99.3%. The material used was polylactic acid (PLA). After printing, the stents were cleaned, disinfected, and finished. Prior to clinical use, CT number calibration confirmed that the relative electron density of the stent material was equivalent to that of soft tissue, thus ensuring no interference with radiotherapy dose calculation and distribution.

### Radiotherapy treatment planning and delivery

Gross tumor volumes and clinical target volumes for all patients were contoured by senior radiation oncologists, while all radiotherapy treatment plans were designed and optimized by senior medical physicists. All patients were treated with volumetric modulated arc therapy (VMAT) using a Varian TrueBeam linear accelerator with 6 MV photon beams. Each plan consisted of two coplanar arcs with complementary gantry rotations (181°–179° and 179°–181°). The prescribed dose was 69–73 Gy in 33–35 fractions (**Table 1**) to the planning target volume (PTV). Treatment planning was performed using the Eclipse treatment planning system (version 16.1), with dose calculation using the Anisotropic Analytical Algorithm (AAA) (version 15.6.06) and optimization using the Photon Optimization (PO) algorithm (version 16.1.2). The calculation grid size was set to 2.5mm. Optimization objectives were set to ensure that at least 95% of the PTV received 95% of the prescribed dose, while dose constraints for organs at risk (OARs) were respected according to the QUANTEC guidelines. Plan evaluation was based on dose-volume histogram (DVH) analysis, conformity index (CI), and homogeneity index (HI).

### CBCT imaging protocol and acquisition of positioning error data

All patients underwent pre-treatment CBCT imaging using the same linear accelerator-integrated system for position verification. For the first two consecutive fractions, CBCT scans were acquired before each fraction to establish baseline setup verification. Thereafter, verification scans were performed once every 5 fractions (equivalent to approximately weekly, given the standard 5-fractions-per-week regimen) for the remainder of the radiotherapy course. The scanning range extended from the cranial vertex to the supraclavicular region, encompassing the whole brain, PGTV, and surrounding bony structures. The acquisition parameters were set as follows: tube voltage, 100 kVp; tube current, 10 mA with automatic dose modulation; field of view (FOV) width, 25–28 cm; FOV length, 18–26 cm; half-arc scan; reconstruction matrix, 512×512; and slice thickness, 2–3 mm. All scans were performed with the patient immobilized in the treatment position.

For offline error extraction, all CBCT images were retrospectively retrieved from the PACS system. For each fraction with CBCT acquisition, each CBCT set was rigidly registered to the corresponding planning CT based on bony anatomy, followed by manual adjustment when necessary to improve soft tissue matching. Manual adjustment was performed when the automatic bone-based registration resulted in a residual mismatch > 3mm at the target-OAR interfaces, as assessed by visual inspection. If the patient’s positioning reproducibility was suboptimal, patient setup was recalibrated to meet the treatment requirements. All CBCT image registrations and positional displacement measurements were independently performed by two trained radiation therapists who were blinded to group allocation. Any discrepancies were resolved by consensus with a third senior investigator. Based on the registration results, positional displacement of the tongue (including posterior, middle, and anterior parts) and parotid glands was recorded in the left–right (LR), superior–inferior (SI), and anterior–posterior (AP) directions. These measurements were used to evaluate interfractional positional variability.

### Acquisition of dosimetric parameters for each organ at risk

Dosimetric parameters for each organ at risk (OAR), including the tongue and parotid glands, were extracted from dose–volume histograms (DVHs) generated from the initial planning CT scans using the Eclipse treatment planning system. The delineation of OARs and the application of dose constraints followed the Quantitative Analysis of Normal Tissue Effects in the Clinic (QUANTEC) guidelines (18). The tongue was specifically delineated according to the contouring guideline for the taste bud bearing tongue mucosa proposed by Spijkerman et al (19). All dose reporting adhered to the recommendations of the International Commission on Radiation Units and Measurements (ICRU) Report No. 83 (20). For each OAR, the following parameters were recorded: Dmin, Dmax, and mean dose (Dmean). These metrics were used to compare dosimetric differences between the two groups.

### Toxicity assessment

Radiation-induced toxicities were assessed using a study-specific structured questionnaire designed to capture patient-reported outcomes in four common head and neck radiotherapy sequelae: dysgeusia, xerostomia, dysphagia, and hearing loss. The questionnaire was developed based on a comprehensive literature review, clinical expertise from two senior radiation oncologists, and the RTOG/EORTC late radiation morbidity criteria, which served as a conceptual reference for symptom definition and grading (**Supplementary Table 2**). The preliminary version was pilot-tested in 10 NPC survivors to assess clarity and face validity, and minor wording modifications were made based on patient feedback.

The final questionnaire covered six clinically relevant domains: hearing and auditory symptoms, gustatory function, xerostomia severity, swallowing function, temporomandibular function and pain, and overall quality-of-life impact. Patients were asked to rate the severity of each symptom during the preceding week on a 5-point Likert-type scale ranging from 0 (none) to 4 (very severe), with higher scores indicating greater symptom burden. All evaluations were conducted via telephone interviews at 12 months after radiotherapy completion by two trained research assistants who were blinded to group allocation, following a standardized script to ensure consistency. The internal consistency of the questionnaire was assessed using Cronbach’s α coefficient, which yielded a value of 0.776, indicating acceptable reliability. Corrected item-total correlations ranged from 0.507 to 0.658, and the α values after deleting each item ranged from 0.680 to 0.759, all lower than the overall α of 0.776, confirming that all four items contributed positively to the scale and were retained for subsequent analyses. The full item composition and detailed structure of the questionnaire are provided in **Supplementary Table 1**.

### Statistical analysis

Statistical analysis was performed using SPSS software (version 26.0, IBM Corp., Armonk, NY, USA). All continuous variables were tested for normality using the Shapiro–Wilk test. For normally distributed data, results were expressed as mean ± standard deviation (SD), and comparisons between the two groups were performed using the independent-samples t-test. For non-normally distributed data, results were expressed as median with interquartile range (IQR), and group comparisons were performed using the Mann–Whitney U test. Categorical variables were compared using the chi-square (χ²) test or Fisher’s exact test, as appropriate. For dosimetric parameters (Dmin, Dmax and Dmean) and positional displacement data (in the LR, SI, and AP directions), group differences were evaluated using the independent-samples t-test or Mann–Whitney U test, depending on the results of normality testing. All statistical tests were two-sided, and a p-value of < 0.05 was considered statistically significant.

## 3 Results

### Patient characteristics

A total of 99 patients were included in the final analysis, with 48 patients in the 3D-printed stent group and 51 patients in the control group. The baseline demographic, clinical, and treatment characteristics of the two cohorts are summarized in **Table 1**. No statistically significant differences were observed between the two groups regarding age (P=0.094), sex (P=0.687), T stage (P=0.105) or N stage (P=0.757). All patients underwent two cycles of induction chemotherapy with paclitaxel and cisplatin (TP), followed by definitive radiotherapy combined with two cycles of concurrent cisplatin as a radiosensitizer. Collectively, these findings indicate that the two cohorts were well-balanced at baseline.

### Radiation-induced toxicities

Compared with the control group, patients receiving individualized intraoral stents exhibited lower frequencies (**Table 2**) and reduced intensities (**Table 3**) of radiation-related toxicities, including dysgeusia, xerostomia, dysphagia, and hearing loss. The stent group also showed lower rates of severe dysgeusia and moderate-to-severe dysphagia (**Tables 2**, **3**). Symptoms of xerostomia, such as nocturnal dryness and frequent water intake, were less frequent in the stent group (**Tables 2**, **3**). No significant intergroup differences were observed for mandibular dysfunction (**Tables 2**, **3**). Exploratory dosimetric data for tongue subregions (integrated into **Tables 2**, **3**) indicated that patients in the stent group received lower doses to the anterior and middle subregions of the tongue compared with controls.

**Table 2:** Comparison of the incidence of radiation-induced toxicities between patients treated with and without individualized 3D-printed intraoral stents.

Group	No. of patients	Xerostomia	Hearing impairment	Dysphagia	Dysgeusia
		Present (n%)	Present (n%)	Present (n%)	Present (n%)
Stent Group	48	48(100%)	16(33.3)	18(37.5)	17(35.4)
Control Group	51	51(100%)	45(88.2)	38(74.5)	42(82.4)
χ²		——	31.54	10.47	20.05
P		——	<0.0001	0.001	<0.0001
Statistical Significance		——	****	***	****

**Table 3:** Comparison of interfractional positional variability of organs at risk between patients treated with and without individualized 3D-printed intraoral stents (mm, mean ± SD).

Organ at Risk (OAR)	Group	Left–Right (LR)	Superior–Inferior (SI)	Anterior–Posterior (AP)
Left	Stent Group	0.119 ± 0.104	0.088 ± 0.073	0.058 ± 0.063
Parotid Gland	Control Group	0.113 ± 0.101	0.114 ± 0.105	0.068 ± 0.065
	U value	52293	47630	49991
	Z value	-1.018	-2.936	-1.976
	P value	0.308	0.003	0.048
	Statistical Significance	——	**	*
Right Parotid Gland	Stent Group	0.116 ± 0.093	0.097 ± 0.080	0.058 ± 0.059
	Control Group	0.113 ± 0.112	0.107 ± 0.062	0.059 ± 0.062
	U value	52838.5	53863.5	53726.5
	Z value	-0.795	-0.377	-0.436
	P value	0.427	0.706	0.663
	Statistical Significance	——	——	——
Left Mandible	Stent Group	0.132 ± 0.100	0.115 ± 0.096	0.061 ± 0.063
	Control Group	0.170 ± 0.236	0.133 ± 0.137	0.068 ± 0.065
	U value	12855	13116	11143.5
	Z value	-0.558	-0.253	-2.578
	P value	0.577	0.8	0.01
	Statistical Significance	——	——	*
Right Mandible	Stent Group	0.111 ± 0.101	0.097 ± 0.087	0.062 ± 0.056
	Control Group	0.141 ± 0.110	0.158 ± 0.080	0.074 ± 0.080
	U value	11141.5	11631.5	12642.5
	Z value	-2.559	-1.993	-0.813
	P value	0.01	0.046	0.416
	Statistical Significance	*	*	——
Posterior Tongue	Stent Group	0.098 ± 0.087	0.130 ± 0.097	0.080 ± 0.078
	Control Group	0.132 ± 0.150	0.149 ± 0.138	0.077 ± 0.086
	U value	48517.5	53821.5	52491
	Z value	-2.568	-0.393	-0.941
	P value	0.01	0.694	0.347
	Statistical Significance	*	——	——
Middle Tongue	Stent Group	0.118 ± 0.138	0.114 ± 0.108	0.077 ± 0.070
	Control Group	0.149 ± 0.140	0.135 ± 0.168	0.087 ± 0.106
	U value	49059.5	52297.5	53173
	Z value	-2.345	-1.019	-0.661
	P value	0.019	0.308	0.509
	Statistical Significance	*	——	——
Anterior Tongue	Stent Group	0.097 ± 0.084	0.120 ± 0.101	0.089 ± 0.078
	Control Group	0.141 ± 0.199	0.153 ± 0.215	0.105 ± 0.099
	U value	45036	51626	51309
	Z value	-4	-1.295	-1.427
	P value	<0.001	0.195	0.154
	Statistical Significance	***	——	——

Data are presented as mean ± standard deviation (SD). Comparisons between the two groups were performed using the independent samples t-test after confirmation of normal distribution by the Shapiro–Wilk test.

### Setup reproducibility and CBCT analysis

**Figure 4:**
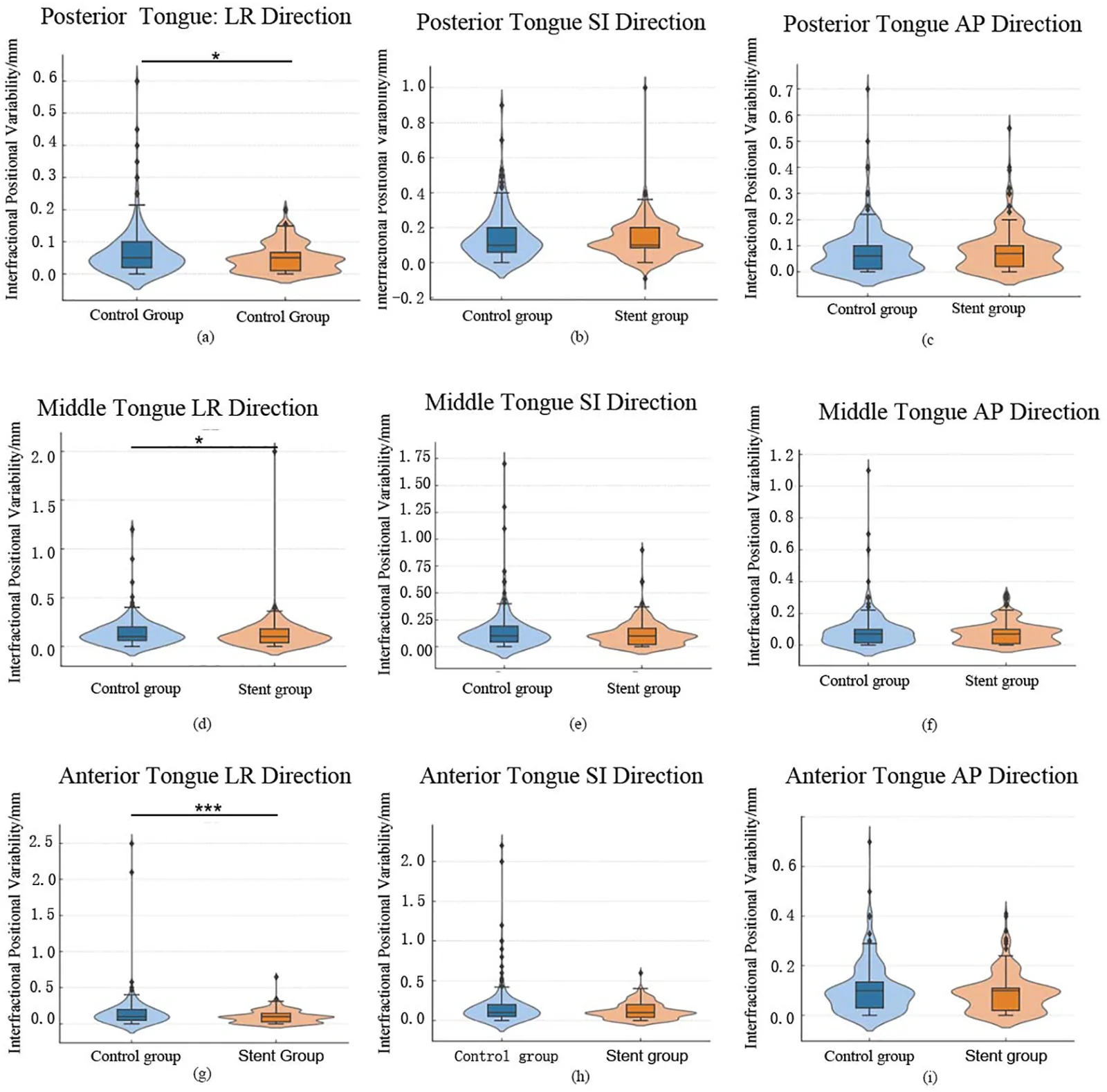
Comparison of interfractional positional variability in the tongue region between the two groups: (a-c) in the Posterior Tongue Region; (d-f) in the Middle Tongue; (g-i) in the Anterior Tongue Region.

CBCT-based analysis demonstrated better setup reproducibility in the stent group than in the control group. Interfractional positional variability of the tongue and parotid glands was reduced with the use of individualized intraoral stents (**Table 4**). The largest reductions in positional shifts were observed for the left/right parotid glands and the anterior and middle subregions of the tongue (**Table 4**; **Figures 4d–i**). For the posterior tongue subregion, the reduction was more modest (**Table 4**; **Figures 4a–c**). The interfractional geometric variation was lower in the stent group across all evaluated subregions.

**Table 4:** Comparison of the severity of radiation-induced toxicities between the stent and control groups one year after radiotherapy.

Group	n	Severity grade, n (%)					Statistic	P value
		0	1	2	3	4		
Xerostomia							U = 370.500	<0.0001
Stent Group	48	0 (0)	15 (31.3)	28 (58.3)	5 (10.4)	0 (0)		
Control Group	51	0 (0)	3 (5.8)	11 (21.6)	16 (31.4)	21 (41.2)		
Hearing impairment							U = 560.000	<0.0001
Stent Group	48	32 (66.7)	12 (25.0)	4 (8.3)	0 (0.0)	0 (0.0)		
Control Group	51	6 (11.8)	11 (21.6)	16 (31.4)	13 (35.5)	5 (9.8)		
Dysphagia							U = 425.000	<0.0001
Stent Group	48	30 (62.5)	11 (22.9)	6 (12.5)	1 (2.1)	0 (0)		
Control Group	51	13 (25.5)	6 (11.8)	13 (25.5)	8 (15.7)	11 (21.6)		
Dysgeusia							U = 560.000	<0.0001
Stent Group	48	31 (64.6)	16 (33.3)	1 (2.1)	0 (0)	0 (0)		
Control Group	51	9 (17.6)	14 (27.5)	13 (25.5)	13 (25.5)	2 (3.9)		

Data are presented as n (%). Group comparisons were performed using the Mann-Whitney U test. Percentages are row percentages (calculated based on the number of patients in each group).

### Dosimetric analysis

Conventional dosimetric parameters, including volume, D2, D98, and Dmean for the evaluated organs at risk between the stent and control groups showed in **Table 5**. Given that the dosimetric data did not follow a normal distribution, non-parametric tests were employed for all comparisons, and results are presented as median with 25th and 75th percentiles (P25, P75). Notably, significant dose reductions were observed in several organs at risk in the stent group compared with the control group. Specifically, the minimum dose (Dmin) to the left inner ear (30.750 [19.875, 37.375] vs. 38.000 [31.350, 47.150] Gy, P=0.013*) and right inner ear (90.800 [65.175, 101.475] vs.100.400 [91.900, 104.350] Gy, P=0.013*) were significantly lower in the stent group. Regarding the left temporomandibular joint, significant reductions were observed in Dmin (37.900 [30.225, 44.250] vs. 42.900 [35.000, 50.200], P=0.010*), Dmax (89.650 [71.000, 94.925[vs. 93.200 [89.500, 97.000], P=0.032*), and Dmean (57.650 [47.525, 66.200] vs 61.600 [56.300, 71.600]., P=0.011*). Additionally, the irradiated volume (72.400 [65.000, 83.800] vs. 92.900 [70.350, 117.300], P=0.016*) and Dmean (46.000 [41.150, 51.200] vs. 53.000 [43.600, 55.900], P=0.032*) of the tongue were also significantly decreased in the stent group. Detailed dosimetric comparisons are summarized in **Table 5**.

**Table 5:** Comparison of the main organ-dose parameters and volumes in the intensity-modulated radiotherapy plans between the stent group and the control group (cGy).

Organ at Risk (OAR)	Parameter	Control group (cGy)	Stent group (cGy)	P Value
optic chiasma	v	1.000(0.300, 1.800)	0.700(0.250, 1.050)	0.037*
	min	21.500(9.175, 37.850)	38.500(11.100, 53.700)	0.084
	max	75.200 (25.150, 80.500)	54.900 (20.100, 76.050)	0.142
	mean	58.600 (16.750, 69.550)	42.450 (12.575, 58.925)	0.113
left eye	v	9.100 (8.200, 10.275)	9.150 (7.825, 9.800)	0.392
	min	4.500 (3.600, 5.150)	4.500 (3.225, 5.350)	0.667
	max	40.800 (29.650, 49.275)	35.550 (21.225, 48.200)	0.185
	mean	11.900 (8.850, 16.675)	12.000 (7.025, 14.950)	0.515
right eye	v	9.050 (7.975, 9.950)	8.650 (7.725, 9.875)	0.354
	min	4.250 (3.200, 5.400)	4.500 (3.425, 5.300)	0.696
	max	38.900 (28.425, 44.100)	33.850 (21.050, 44.800)	0.301
	mean	12.150 (8.975, 17.025)	11.350 (6.825, 14.300)	0.185
inner ear-L	v	2.200 (1.500, 2.900)	2.050 (0.300, 3.225)	0.568
	min	38.000 (31.350, 47.150)	30.750 (19.875, 37.375)	0.013*
	max	100.400 (91.900, 104.350)	90.800 (65.175, 101.475)	0.013*
	mean	68.000 (57.500, 79.500)	53.250 (43.175, 61.825)	0.003**
inner ear-R	v	2.300 (1.400, 2.850)	2.100 (0.300, 3.125)	0.257
	min	34.200 (29.050, 44.850)	30.800 (23.550, 38.875)	0.108
	max	96.800 (90.400, 101.900)	81.200 (64.375, 92.325)	0.003**
	mean	63.800 (52.850, 73.450)	47.550 (44.000, 58.575)	<0.001***
Lens-L	v	0.200 (0.100, 0.200)	0.200 (0.100, 0.225)	0.188
	min	6.350 (4.925, 7.700)	6.150 (4.975, 7.425)	0.486
	max	8.850 (6.775, 10.525)	8.900 (7.375, 10.175)	0.879
	mean	7.450 (5.725, 9.100)	7.100 (6.275, 8.525)	0.644
Lens-R	v	0.200 (0.100, 0.200)	0.200 (0.100, 0.200)	0.140
	min	6.500 (5.100, 7.500)	6.250 (4.900, 7.400)	0.561
	max	8.800 (6.800, 10.600)	8.900 (7.200, 10.000)	0.837
	mean	7.500 (5.800, 8.700)	7.300 (5.875, 8.400)	0.718
mandible-L	v	56.550 (37.000, 68.025)	46.500 (31.400, 64.100)	0.326
	min	21.750 (16.925, 25.650)	20.400 (17.400, 23.000)	0.360
	max	99.550 (97.300, 101.900)	100.000 (96.100, 103.200)	0.493
	mean	54.750 (49.725, 61.375)	53.600 (47.400, 61.000)	0.690
mandible-R	v	34.900 (30.600, 40.200)	33.900 (29.425, 39.375)	0.637
	min	23.700 (15.600, 25.200)	20.200 (16.975, 23.075)	0.483
	max	102.100 (96.725, 103.650)	98.200 (90.700, 101.000)	0.015*
	mean	53.500 (44.200, 61.300)	56.400 (47.975, 64.450)	0.535
Optic nerve-L	v	0.500 (0.300, 0.800)	0.500 (0.375, 0.800)	0.577
	min	14.550 (7.225, 26.400)	12.000 (8.025, 30.225)	0.949
	max	73.900 (21.775, 77.875)	71.000 (26.675, 76.000)	0.475
	mean	36.200 (11.525, 50.900)	32.950 (14.050, 56.175)	0.952
Optic nerve-R	v	0.450 (0.300, 0.800)	0.500 (0.400, 0.700)	0.601
	min	15.400 (7.050, 27.675)	11.900 (8.150, 27.850)	1.000
	max	65.950 (23.375, 78.575)	68.900 (24.950, 75.550)	0.471
	mean	37.950 (12.125, 57.975)	32.200 (13.350, 52.000)	0.468
Parotid-L	v	21.150 (16.625, 31.600)	21.600 (16.250, 28.150)	0.477
	min	14.500 (12.200, 17.250)	13.500 (11.500, 16.200)	0.122
	max	101.800 (98.375, 104.875)	100.200 (96.900, 103.850)	0.398
	mean	49.200 (44.150, 55.075)	46.200 (42.800, 52.600)	0.077
Parotid-R	v	21.750 (17.275, 30.800)	20.000 (15.850, 25.800)	0.218
	min	14.350 (11.625, 16.100)	13.800 (11.550, 16.650)	0.762
	max	100.350 (93.800, 103.575)	99.900 (96.250, 105.000)	0.336
	mean	49.650 (38.350, 53.400)	48.800 (42.250, 53.100)	0.972
Tmjoint-L	v	2.100 (1.100, 3.000)	2.400 (1.375, 3.000)	0.504
	min	42.900 (35.000, 50.200)	37.900 (30.225, 44.250)	0.010*
	max	93.200 (89.500, 97.000)	89.650 (71.000, 94.925)	0.032*
	mean	61.600 (56.300, 71.600)	57.650 (47.525, 66.200)	0.011*
Tmjoint-R	v	2.000 (1.125, 3.200)	2.350 (1.650, 3.175)	0.291
	min	39.350 (33.250, 45.200)	38.850 (27.375, 45.950)	0.509
	max	90.200 (79.525, 95.175)	86.000 (73.750, 92.625)	0.173
	mean	59.950 (53.850, 64.450)	55.600 (49.050, 62.925)	0.137
Tongue	v	92.900 (70.350, 117.300)	72.400 (65.000, 83.800)	0.016*
	min	23.700 (18.400, 28.400)	19.500 (13.150, 25.550)	0.178
	max	99.300 (90.000, 105.000)	98.000 (90.650, 101.400)	0.303
	mean	53.000 (43.600, 55.900)	46.000 (41.150, 51.200)	0.032*

Normality was assessed using the Shapiro–Wilk test, the dosimetric and volumetric parameters for organs at risk were found to be non-normally distributed. Data are presented as median (P25, P75). Between-group comparisons were performed using the Mann–Whitney U test. P < 0.05 was considered statistically significant.

## 4 Discussion

The main finding of this study was that patient-specific 3D-printed intraoral stents were associated with significantly improved interfractional setup reproducibility for mobile organs at risk, particularly the tongue and parotid glands, in patients with NPC undergoing VMAT, and this geometric benefit was correlated with clinically meaningful reductions in radiation-induced toxicities, including taste disturbance, dysphagia, and hearing loss. These results support our hypothesis that enhanced geometric stabilization during treatment delivery may reduce unintended radiation exposure to functional substructures.

Another key contribution of this study lies in our subregional analysis of the tongue. Rather than treating the tongue as a homogeneous organ, we stratified it into anterior (tip), middle, and posterior (base) compartments based on distinct anatomical and functional characteristics. This distinction is clinically meaningful, the well-recognized differences in embryological origins, neural innervation, and physiological roles across these regions (21). The intraoral stent conferred greater stabilization to the anterior and middle tongue than to the posterior region. This regional gradient likely reflects the anatomical anchoring of the tongue base to adjacent pharyngeal structures. Clinically, this pattern may explain why taste preservation—largely mediated by the anterior and middle tongue—was superior in the stent group, whereas swallowing outcomes, which depend more on posterior tongue mobility, showed less improvement (**Table 4**). In addition, the absolute cranio-caudal displacement range of mandible was significantly narrower in the stent group (**Supplementary Figure 2**), while the average mean displacement value showed no statistical inter-group difference (**Table 3**). The statistical non-significance of mean offset does not negate the stent’s effect of reducing positional fluctuation range.

Mechanistically, the improvement in lingual functions, including taste, may be attributable to two synergistic factors: stabilization of tongue position to minimize inter-fraction setup errors, and posterior displacement of the tongue to reduce its projection area within the lateral opposed radiation fields (**Supplementary Figure 1**). Despite these geometric improvements, conventional static dosimetric parameters for the whole tongue and parotid glands (including D_2_, Dmean, and D_98_) showed no significant differences between groups. This apparent discrepancy—improved functional outcomes without corresponding changes in dose–volume parameters—may be explained by the fact that the primary benefit of the stent lies in reducing inter-fractional anatomical variability rather than altering nominal planned doses. Static plan-based dosimetry may not fully capture the cumulative impact of anatomical changes during treatment, and thus geometric reproducibility across fractions may represent an additional determinant of toxicity beyond conventional parameters. Therefore, the functional benefits observed in the stent group likely reflect not a reduction in planned dose per se, but rather a more reliable translation of the planned dose into the actual delivered dose across fractions, achieved through enhanced positional stability and favorable projection geometry. An exception to this pattern, however, was observed for the inner ear, as discussed below.

Notably, in contrast to the findings for the whole tongue and parotid glands, our *post-hoc* dosimetric analysis focusing on the inner ear revealed significantly lower inner ear doses (including Dmean and/or Dmax) in the 3D-printed stent group compared with the control group. Previous dosimetric studies and clinical guidelines for radiotherapy in locally advanced nasopharyngeal carcinoma have recommended limiting the inner ear mean dose to approximately 44–50 Gy; however, the risk of sensorineural hearing impairment remains clinically significant even when these constraints are respected. The observed dose reduction to the inner ear provides a plausible dosimetric explanation for the preservation of hearing function in the stent group, suggesting that the geometric stabilization afforded by the stent may contribute to more consistent dose delivery to the inner ear region while reducing incidental exposure.

Taken together, the contrasting dosimetric patterns between the inner ear (significant dose reduction) and the mobile OARs such as the tongue and parotid glands (no significant dose reduction) suggest that the mechanisms underlying clinical benefit may vary among anatomical structures. We propose a dual-mechanism framework: for relatively fixed structures such as the inner ear, the benefit of the stent is more directly reflected as dose reduction (a direct sparing effect), potentially resulting from improved reproducibility of patient positioning and beam geometry. For mobile organs such as the tongue and parotid glands, the primary contribution involves improved geometric stability and reduced uncertainty in delivered dose (an indirect stabilization effect) rather than a reduction in planned dose parameters. This framework reconciles our dosimetric and geometric findings and suggests that toxicity reduction in NPC radiotherapy may operate through two complementary pathways.

Previous systematic reviews have suggested that intraoral stents may reduce radiation exposure to healthy oral tissues, yet definitive evidence of their clinical benefit remains limited (**Supplementary Table 3**). Compared with conventional non-customized stents, our 3D-printed patient-specific devices improved inter-fraction setup reproducibility, reduced parotid positional variation, and yielded better patient-reported outcomes. More importantly, while earlier studies largely attributed stent benefits to physical displacement of normal tissues away from radiation fields (22), our findings suggest that improved geometric reproducibility may represent an equally important—and potentially more critical—mechanism in nasopharyngeal carcinoma radiotherapy. This distinction may explain the differences between our dosimetric results and those previously reported. From a clinical standpoint, patient-specific stents offer a simple, cost-effective means of mitigating treatment-related toxicities. Moreover, the enhanced anatomical stability they provide opens up new possibilities for future applications in online adaptive radiotherapy and individualized treatment optimization.

Several limitations should be acknowledged. First, this was a retrospective, single-center study with a relatively limited sample size; nevertheless, the comparable baseline characteristics between groups and the consistency of the observed effects across multiple endpoints support the robustness of our findings. Second, toxicity outcomes were collected at a single post-treatment time point rather than through longitudinal prospective evaluation; future studies should incorporate serial assessments to capture the temporal evolution of symptom recovery. Third, although interfractional setup errors were evaluated using daily CBCT, cumulative delivered dose reconstruction was not performed; future investigations using deformable image registration-based dose accumulation are warranted to validate our proposed mechanism. Fourth, although we performed a subregional analysis of tongue motion and functional outcomes, formal dosimetric comparison for the anterior, middle, and posterior tongue subregions was not performed, as the standard clinical contouring protocol (RTOG guidelines) defines the tongue as a single organ at risk, and retrospective re-contouring of all cases with precise subregional delineation was not feasible. Nevertheless, the observed functional gradient—superior taste preservation versus less improvement in swallowing—is consistent with the geometric differences we demonstrated. Future studies with pre-planned subregional tongue delineation are warranted to validate the dose–function relationship. Despite these limitations, the convergence of geometric, dosimetric, and clinical evidence provides a coherent narrative supporting the clinical utility of patient-specific intraoral stents in NPC radiotherapy.

Collectively, our findings underscore the value of considering interfractional anatomical variability as a determinant of treatment-related toxicity beyond conventional static dosimetric parameters. The convergence of geometric, dosimetric, and patient-reported evidence in this study supports the integration of patient-specific 3D-printed intraoral stents into routine clinical practice for NPC radiotherapy, and the dual-mechanism framework proposed herein provides a testable hypothesis for future investigations.

## 5 Conclusions

This study demonstrates that patient-specific 3D-printed intraoral stents improve interfractional setup reproducibility for mobile organs at risk, particularly the tongue and parotid glands, during nasopharyngeal carcinoma radiotherapy. Although conventional plan-based dosimetric parameters for the parotid glands and whole tongue were not significantly different between groups, patients receiving stents experienced clinically meaningful reductions in taste disturbance and swallowing difficulty, together with improved hearing preservation supported by lower inner ear dose parameters.

These findings suggest that the benefits of intraoral stents may arise through two complementary mechanisms: direct dose reduction for relatively fixed organs at risk, such as the inner ear, and improved delivery consistency through enhanced geometric stabilization for mobile structures, such as the tongue and parotid glands. Unlike previous studies that mainly attributed intraoral stent benefits to physical tissue displacement and dose reduction, our findings highlight the potential importance of reducing interfractional anatomical variability in nasopharyngeal carcinoma radiotherapy.

Despite the limitations of this retrospective single-center study, these results support the clinical feasibility of customized 3D-printed intraoral stents as an adjunctive approach during VMAT. Dose reconstruction on CBCT images or accumulated dose analysis was not performed in this study, which is consistent with the common practice in similar retrospective dosimetric studies in the field. Nevertheless, to validate the mechanistic hypothesis proposed in this study—that improved geometric stability reduces dose uncertainty—future investigations are warranted to perform dose accumulation on the actual patient anatomy using deformable image registration techniques.

Collectively, our findings underscore the value of considering interfractional anatomical variability as a determinant of treatment-related toxicity beyond conventional static dosimetric parameters. The convergence of geometric, dosimetric, and patient-reported evidence in this study supports the integration of patient-specific 3D-printed intraoral stents into routine clinical practice for NPC radiotherapy, and the dual-mechanism framework proposed herein provides a testable hypothesis for future investigations. Further prospective studies incorporating accumulated dose verification and longitudinal toxicity assessment are needed to confirm these mechanistic interpretations and determine their broader clinical applicability.

## Data Availability

The original contributions presented in the study are included in the article/**Supplementary Material**. Further inquiries can be directed to the corresponding authors.
